# Here, There, and Everywhere: The Ubiquitous Distribution of the Immunosignaling Molecule Lysophosphatidylcholine and Its Role on Chagas Disease

**DOI:** 10.3389/fimmu.2016.00062

**Published:** 2016-02-19

**Authors:** Mário Alberto C. Silva-Neto, Angela H. Lopes, Georgia C. Atella

**Affiliations:** ^1^Programa de Biologia Molecular e Biotecnologia, CCS, Instituto de Bioquímica Médica Leopoldo de Meis, Universidade Federal do Rio de Janeiro, Rio de Janeiro, Brazil; ^2^Centro de Ciências da Saúde, Instituto de Microbiologia Prof. Paulo de Góes, Universidade Federal do Rio de Janeiro, Cidade Universitária – Ilha do Fundão, Rio de Janeiro, Brazil

**Keywords:** lysophosphatidylcholine, Chagas disease, nitric oxide, MAPK, *Rhodnius prolixus*, macrophage

## Abstract

Chagas disease is a severe illness, which can lead to death if the patients are not promptly treated. The disease is caused by the protozoan parasite *Trypanosoma cruzi*, which is mostly transmitted by a triatomine insect vector. There are 8–10 million people infected with *T. cruzi* in the world, but the transmission of such disease by bugs occurs only in the Americas, especially Latin America. Chronically infected patients will develop cardiac diseases (30%) and up digestive, neurological, or mixed disorders (10%). Lysophosphatidylcholine (LPC) is the major phospholipid component of oxidized low-density lipoproteins associated with atherosclerosis-related tissue damage. Insect-derived LPC powerfully attracts inflammatory cells to the site of the insect bite, enhances parasite invasion, and inhibits the production of nitric oxide by *T. cruzi*-stimulated macrophages. The recognition of the ubiquitous presence of LPC on the vector saliva, its production by the parasite itself and its presence both on patient plasma and its role on diverse host × parasite interaction systems lead us to compare its distribution in nature with the title of the famous Beatles song “Here, There and Everywhere” recorded exactly 50 years ago in 1966. Here, we review the major findings pointing out the role of such molecule as an immunosignaling modulator of Chagas disease transmission. Also, we believe that future investigation of the connection of this ubiquity and the immune role of such molecule may lead in the future to novel methods to control parasite transmission, infection, and pathogenesis.

## Introduction

Lysophosphatidylcholine (LPC) is a major regulator of several biological processes. It is produced by the hydrolysis of the fatty acid at sn-2 position of phosphatidylcholine (PC) catalyzed by phospholipase A2 (PLA2) or alternatively by its transfer from PC to cholesterol by the action of another enzyme a cholesterol acetyltransferase (1–3). Such molecule was originally believed to play a role exclusively on membrane structure but their involvement on the pathogenesis of several human diseases is increasingly clear in the last few years. Its recognition as a causative factor has occurred by mainly by two different set of data. The first line of evidence toward a functional and signaling role of LPC is due to the close link between plasma lipid profile and pro-oxidant reactions common in inflammatory diseases, especially in atherosclerosis. The second route of evidences lies on the effects of LPC on cells involved in innate immunity. We will discuss the growing of such concepts and how they overlap and support the role of such lysophospholipid in parasitic diseases as follows.

Lysophosphatidylcholine and the bioactive lipids lysophosphatidic acid (LPA), sphingosine-1-phosphate (S1P), and sphingosylphosphorylcholine (SPC) modulate a wide number of biological processes on mammalian cells. LPC has been classically involved in various physiological events and is already known as a central molecule in various pathological conditions but is especially present during the deposition and infiltration of inflammatory cells and deposition of atheroma, as discussed above (4–6). Research aimed at LPC increased substantially from the fact that these molecules are involved in atherosclerosis (7). The idea that several phospholipases secreted by circulating leukocytes may participate in this pathology was soon proposed. Thus, the current model suggests that diabetes and hypercholesterolemia contribute to generate a large number of low-density lipoprotein (LDL) particles in plasma, which can undergo oxidation of unsaturated fatty acids, generating an oxidized particle (ox-LDL). Since on average, 50% of LDL fatty acids are arachidonic acid and linoleic acid, the chances of an oxidative event like this are huge. OxLDL is a potential cause of increased expression of inflammatory markers. TNF-α, MCP-1, and MCSF expression are increased by oxLDL and attract and differentiate monocytes to the lesion site. Accordingly, LPC is a powerful chemoattractant molecule to macrophages. It is also generated by cells during apoptosis, as mentioned above. oxLDL particles are recognized by PLA2 secreted by different cells in plasma, including the types IIa, V, and X. In conclusion, plasma LPC is derived from the hydrolysis of PC mainly present in LDL and cell membranes by several different subfamilies of PLA2s usually following and oxidative event on its fatty acids.

*Trypanosoma cruzi* infects the vertebrate host through skin bites produced by bugs during their feed or by interaction with conjunctival mucosa. This interaction sometimes produces the Romaña signal or chagoma whose histology is defined by a large number of mononuclear cells (8). It is likely that *T. cruzi* stimulates skin cells to produce mediators that trigger a local inflammatory response. Chagas disease displays an acute phase, followed by a chronic phase where the parasites are physically linked to inflammation sites in the heart and esophagus (9–15). The disease is considered today as multifactorial once pathogen and host are continuously interacting throughout the whole patient life (16). Following the first 2–3 weeks of the vector bite, *T. cruzi* infection is manifested by a high load of parasites in the blood and tissues. Acute infection is characterized by a huge activation of the immune system. Such activation includes the production of high levels of cytokines, a large activation of T and B cells, lymphadenopathy, and splenomegaly. Also, it displays a visible inflammation due to the infection in tissue niches. The acute phase leads to the development of acquired immunity. Such mechanism ensures the effective control of parasitemia. The chronic phase takes place throughout the patient’s life and is associated with low levels of parasite in the host. The beginning of chronic infection in Chagas disease is asymptomatic in most patients. However, clinical manifestations will range from no symptoms to the involvement of the cardiovascular symptoms and/or gastrointestinal complications as the disease progresses (17, 18). The innate immune system appears to be essential for at least two important aspects of Chagas disease: control of parasite replication in host tissue and the progress of the inflammatory reaction. The inflammatory reaction itself may be a major cause of tissue damage and dysfunction of certain organs in the patient (18). Experiments in *T. cruzi* infection models have shown that a potent Th-1 CD4 and CD8 immune response controls parasitemia during the initial stage of the disease. Such immune response is characterized by the production of IFN-γ, TNF-α, and IL-12, as well as the production of nitric oxide (NO). Additionally, cells of innate immunity, such as “natural killer” (NK) cells, dendritic cells, and macrophages, are also key factors in the initial control parasite replication (11, 17, 19).

In recent years, research on Chagas disease has focused on the investigation of the role of pathogen-associated molecular patterns (PAMPs) protozoa, which are the targets of the innate immune response receptors. Also the problem of identification of relevant receptors for the innate immunity to the parasite during the course of the disease in the host has been addressed by several laboratories. This strategy ultimately aimed at developing therapeutic interventions through the use of derivatives of PAMPs present in the parasites. Glycosyl-phosphatidylinositol (GPI) is the name given to the first glycoconjugate identified in *Trypanosoma brucei*. This molecule was identified with the function of anchoring proteins on the cell surface (20–23). *T. cruzi* express on their surface glycoproteins anchored by GPIs (20–23). GPI anchors from trypomastigotes include mucin-like glycoproteins (mucin-GPI) and are key molecules responsible for stimulating the host’s immune system (24–27). These mucin-GPIs activate macrophages and lead to the synthesis of proinflammatory cytokines, chemokines, and NO (28). The innate immune response to *T. cruzi* infection largely results from the activation of signaling pathways triggered by Toll-like receptors (TLRs).

Toll-like receptors recognize conserved structural motifs present on different pathogens. Following their activation, TLRs trigger intracellular signaling cascades that build up the host immune response (18, 29). There are 10 TLRs described in human and 12 in mice (18, 29). GPI-induced stimulus occurs during the initial phase of infection, where macrophages respond to trypomastigotes in a TLR-dependent mechanism. At this point in time the production of IL-12, TNF-α and the activation of CD4 and CD8 responses through IFN-γ will take place (30). TNF-α and IFN-γ-activated macrophages seems to have an important role in the control of parasite growth. Anchors free of proteins or GPI or glicoinositol phospholipids (GIPLs) are also able to stimulate the host immune system. GIPLs are similar to GPIs but contain ceramide in their lipid portion (24, 25). TLRs 2, 4, and 9 are the main TLRs involved in the innate immune response to *T. cruzi* (24, 31–36). TLR2 was found to be activated by mucin-GPI (24, 31–36). The heterodimer composed of TLR2 and TRL6 receptor is activated by GPI mucin and its co-receptor CD14 (34). *T. cruzi* GIPL activates the inflammatory response *via* TLR4. It induces neutrophil recruitment to the peritoneal cavity of mice and this effect is partly dependent on IL-1β production (32, 36). The *T. cruzi* genomic DNA also plays an important role in the vertebrate host proinflammatory response. Finally, TLR 9 is activated by CpG motifs in non-methylated DNA (34, 35). In the innate immune response mediated by TLRs, *T. cruzi* can also stimulate a TLR-independent response leading to the production of IFN-β and IFN-γ. This is due a rise on intracellular calcium concentration that activates calcineurin and calmodulin-dependent proteins (37–39).

## Here: LPC-Mediated Handling of Innate Immunity at the Site of Parasite Infection

**Figure 1:**
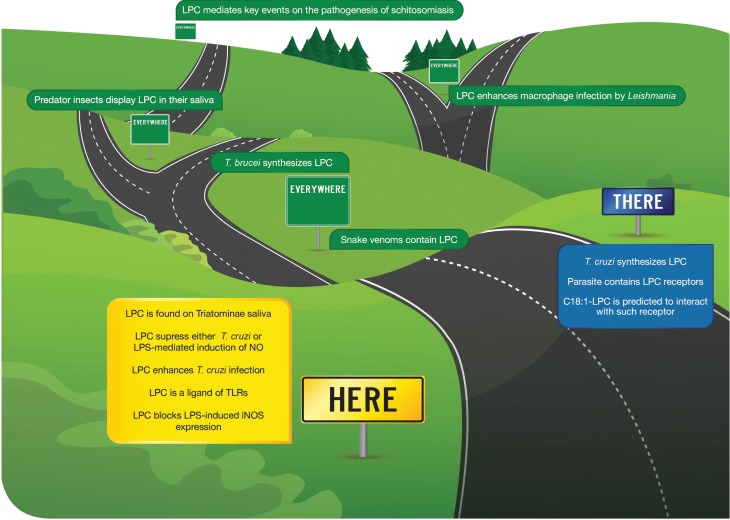
**Here, there, and everywhere**. This cartoon depicts the major findings in recent years regarding the role of LPC on infectious diseases. Here, originally found as a vector, salivary phospholipid able to modulate Chagas disease through the suppression of NO production. There, LPC was recently described as the product of parasite synthesis. Everywhere, several reports have involved LPC on different aspects of parasitic diseases but also a relevant mediator molecule used to subvert the prey by hunting insects. Finally, its role as a major component of snake venom is also shown.

Our group demonstrated, for the first time, the presence of phospholipids and lysophospholipids in saliva and stool hematophagous organism *Rhodnius prolixus*, a triatomine vector of Chagas disease (40, 41). Such finding engaged salivary LPC into the host plasma environment and uncovered its role as both an anti-hemostatic and immunomodulatory molecule. The main lipids present in the saliva of *R. prolixus* are PC and LPC (40, 41). Salivary LPC is an additional anti-hemostatic molecule that is part of the pharmacological arsenal injected into the bite site to allow the insect feeds. It inhibits platelet aggregation and increases the production of NO in endothelial cells. Thus, the LPC was originally described as a molecule with antiplatelet and vasodilatory activity, and few years later, its effect as an immunomodulator of *T. cruzi* infection has been demonstrated (40, 41) (Figure 1).

The role of LPC as a modulator of *T. cruzi* infection is caused by three main mechanisms summarized as follows:

1. LPC is a vector-derived molecule. Following attraction of inflammatory cells to the insect bite site, it enhances the chance of infection by the latter arrival of *T. cruzi* (40, 41).
2. LPC produces a surge on intracellular calcium concentrations in macrophages. This will also enhance parasite invasion. It also modulates the phosphorylation of key signaling molecules such as mitogen-activated kinases (MAPKs) (41, 42).
3. LPC inhibits the production of NO by *T. cruzi*-stimulated macrophages, lipopolysaccharide (LPS), or LPS in the presence of IFN-γ. Thus, it handles the immune system of the vertebrate host (41, 42).

Such findings implied that LPC is a multifactorial signaling molecule, with signaling, anti-hemostatic, and immune effects on the context of Chagas disease transmission (43–46). The establishment of infection by *T. cruzi* depends on the initial invasion of these cells by the parasite (47–49). This leads to the assumption that the salivary LPC can facilitate parasitic infection, favoring not only insect feeding but also the preparation of macrophage susceptibility for the arrival of the parasite, minutes or hours after the initial bite. Salivary LPC injection into the host’s skin followed by inoculation of the parasite leaded to an increase of threefold to sixfold on blood parasitemia (41). LPC effect on parasitemia relies on the activation of macrophage chemotaxis and suppression of NO synthesis. We also show an increase in parasite loads within macrophages induced by either 500-fold diluted saliva or LPC. Such results were the first demonstration of a factor able to potentiate the transmission of Chagas disease and the first implication of a lysophospholipid as a modulator of an infectious disease (41, 42).

Activation of TLRs on host cells and the production of TNF-α, IL-12, and NO, required the adapter protein MyD88 (20–24, 30). TLRs 2, 4, and 9 have been implicated in the control of *T. cruzi* infection (20–24, 30). The involvement of TLR2 in the interaction between the parasite and macrophages was also demonstrated (24, 30). TLR2 expression is essential for the induction of IL-12, TNF-α, and NO. This receptor is activated by the parasite molecules, such as GPI anchors, that are isolated from the surface trypomastigotes of *T. cruzi*. Curiously, IL-12 synthesis in macrophages exposed to *T. cruzi* is not affected by saliva (41). Interestingly, in bone marrow derived macrophages derived from TLR2-deficient mice IL-12 production is largely suppressed by LPC (41). These data indicate that in some types of cells this cytokine production can be affected by LPC through a TLR2-independent mechanism.

Furthermore, *T. cruzi* GIPLs are TLR4 agonists with proinflammatory effects (32, 36). We have shown that NO production induced by the parasite or LPS, another ligand of TLR4 or in peritoneal murine macrophages or bone marrow-derived macrophages is blocked in both cases, the LPC even in the presence of IFN-γ vitro (41). The LPC’s ability to reverse the induction of NO production in every case, almost regardless of the type of ligand suggests that this bioactive lipid should act on a single pathway. Usually, LPC-sensitive receptors exhibit remarkable overlap in their specificity to the ligands they interact with and vice versa. Different receptors have been proposed for LPC. This includes G2A a G protein-coupled receptor, and GPR4, another important candidate (50). It is noteworthy the low reproducibility of LPC studies where radioactive lipids were used. Together with the capacity of G2A to bind to fatty acids and protons, such findings brought a high complexity to this field (51). Thus, G2A is still recognized as the most known receptor to convey signals mediated by LPC presence in the chemical environment of cells. The redistribution of G2A receptor itself and the exposure of TLR4 on cell surface are highly influenced by LPC metabolism (51, 52). In this latter case, intracellular LPC amount is under control due to the activity of LPC-acyltransferase (LPCAT). This enzyme uses LPC to produce phospholipids. The incubation of monocytes with LPS activates the enzyme and increases the transport of TLR4 to cell-membrane rafts (52). The LPCAT inhibitor, thiophenyl 5-hydroxyethyl 5′3 pyridine (HETP), increases the ratio LPC/PC while it reverses the effect of LPS. Thus, *T. cruzi* may promote a redistribution of G2A and TLR4 on cell surface during infection. This should be investigated in the future by both proteomic and immunological approaches.

In apoptotic cells, LPC is generated by a PLA2- calcium-independent activated caspase-3. LPC then attracts phagocytes to cells and represents a sign of recognition of ongoing apoptosis (50, 53, 54). LPC-induced chemotaxis in Chagas’ disease is intriguing once the uptake of apoptotic cells by macrophages infected with *T. cruzi* parasite stimulates their growth (55). Furthermore, the infective stages of *T. cruzi* are capable of generating lipid messengers, including LPC, that modulate the signaling of the host cell (56). LPC activates some protein kinase C isoforms (57). It is probably that different PKC isoforms are activated in different cell types. Also, when combined with different types of TLRs and adapters, the LPC-mediated signaling repertoire may produce a specific and yet to be studied particular mechanism of immunosuppression (Figure 1).

In order to evaluate some of these questions, we have addressed the role of LPC on TLR-mediated signaling pathway using HEK 293A cells. Such cells were transfected with TLRs constructs and stimulated with LPCs displaying different fatty acid chain lengths and saturation levels (42). All tested LPCs activated both TLR4 and TLR2-1 signaling pathways. Such results were confirmed through the evaluation of NF-κB activation and IL-8 production. Similar results were obtained when using peritoneal murine macrophages. These cells responded to LPC stimulation by displaying NF-κB translocation. Curiously, when incubated in the presence of LPS, LPC counteracted several features of TLR4 signaling. In this case, NF-κB translocation, NO synthesis, and the expression of inducible nitric oxide synthase (iNOS) were blocked. Such phenotypes occur concomitantly with a hierarchical activation of the MAPKs p38 and JNK, but not ERK, in murine macrophages. Also, LPC blocked LPS-induced ERK activation in peritoneal macrophages but not in TLR-transfected cells. Such results indicated that LPC behaves as a proinflammatory molecule in the absence of traditional TLR ligands. However, when LPS and LPC are present at the same time, a partial silencing of the canonical TLR4 pathway takes place. Such silencing involves the downregulation of ERK pathway and does not affect p32 and JNK. Under these conditions, LPC assumes an anti-inflammatory phenotype through yet unknown ligands on cell surface (42).

## There: LPC is Synthesized by Trypanosomatid Parasites

Phosphatidylcholine and LPC are synthesized by *T. cruzi* (58, 59), *T. brucei* (60), *Leishmania* spp. (61, 62), as well as by the malaria parasite, *Plasmodium falciparum* (63). Intriguingly, more than 50% of the total lipids secreted by *T. cruzi* were identified as PC and LPC (58). Recently, we have purified and structurally characterized a C18:1-LPC in *T. cruzi*, which present a platelet-activating factor (PAF)-like activity, as it aggregates rabbit platelets (59). Comparable to PAF, platelet aggregation was completely blocked by the PAF receptor antagonist, WEB 2086. Considering that increased platelet aggregation related to myocarditis is observed in Chagas disease, it is possible that C18:1-LPC is an important lipid mediator in the progression of this disease. Nevertheless, what functions endogenous *T. cruzi* LPC have in the infection is yet to be unveiled. Such findings enhance our view that during the complete cycle of trypanosomatids transmission LPC will be able to handle surrounding cells, therefore enhancing parasite survival (Figure 1).

## Everywhere: Role of LPC in Other Parasitic Infections

In recent years, our group and others have explored the role of lysophospholipids, and especially LPC on the transmission and pathogenesis of different parasitic diseases. Also, it was demonstrated that LPC plays a role on prey × predator. Such models lead us to the view that LPC is a ubiquitous modulator of host–parasite interaction (Figure 1).

### Leishmaniasis

A key step on *Leishmania* infection is the invasion of macrophages, which usually occurs through an endocytic process. Such process leads to the formation of the parasitophorous vacuole (PV) that will harbor the parasite. PV was isolated from *Leishmania*-infected macrophages, and the analysis of its phospholipid composition was conducted (64). The levels of LPC increased as compared with non-infected cells. Such results were obtained by allowing either the promastigote or amastigote forms of the parasite to interact with macrophages previously labeled with radioactive phosphate. Thus, these findings indicate that following invasion there is a remodeling of host cell phospholipids, which enhances the production of LPC. The results obtained in the *T. cruzi* model (41, 42), where LPC was pointed out as a negative modulator of NO production suggest that *Leishmania*-induced remodeling of PV may handle the microbicide production and protects the parasite. If this mechanism is essential to allow parasite escape from PV should be investigated in the future. It is important to consider that the use of miltefosine, the first orally active anti-leishmanial drug when directly used to treat *Leishmania donovani* promastigotes, induced at higher doses the increase on LPC production on total parasite membrane extract, probably due to the activation of PLA2s (65). Curiously, such effect is confined to some specific membranar structure of the parasite since it is lost when the plasma membrane is evaluated separately. The involvement on LPC on *Leishmania*-infected was recently busted by the finding that such molecule in BALB/c mouse-derived DC infection (66). LPC enhanced by 92% the infection index was correlated to a delay on cell maturation as evaluated by the expression of CD86^+^/CD11c^+^. Treatment of such cells with LPS displayed the opposite effects. Also, the authors found that LPC reversed the immune balance between IL-10, TNF-α, and IL-6 once it induced a huge increase on the first cytokine while not affecting of slightly reducing the proinflammatory ones (66). Finally, they observed that LPC is also able to selectively increase arginase1 expression by 395%. This enzyme is commonly induced by Th2 cytokine response set and enhances parasite proliferation. Such effect is paralleled by a suppression of iNOS activation, which keeps the levels of the NO quite low in *Leishmania*-infected cells. Indoleamine 2,3-dioxygenase (IDO) catalyzes tryptophan catabolism through kynurenine pathway. The depletion of tryptophan blocks parasite proliferation. *Leishmania* and LPC induced IDO activation, which usually leads to the accumulation of kynurenine metabolites blocking Th1 response. Also, the increase on arginase expression and downregulation of iNOS production depletes invaded cells of GSH, which induced acute oxidative stress. LPC has recently been described as a major inducer of macrophage polarization toward the M2 phenotype (67–69). In monocytes obtained from patients with Post Kala-Azar Dermal Leishmanisis (68), it was noticed a strong downregulation of TLR2/4 receptors and an increased expression of PPAR-γ and arginase-1. Also, IL-4, IL-10, and IL-13 were significantly raised as compared to healthy individuals. Finally, it increased classical M2 markers CD206, arginase-1, and PPARG in monocytes. Such findings strongly demonstrate that M2 polarization of macrophages is a hallmark of *Leishmania* infection *in vivo* and suggest that a bridge to the LPC-induced effects is the next step toward the development of drugs that modulate disease chronicity. These drugs should be anti-leishmanial molecules that ensure the repolarization of M2 macrophages toward the M1 proinflammatory and protective phenotype (68).

### Human African Trypanosomiasis

A global metabolic profiling of plasma of patients carrying *T. brucei rhodesiense* [human African trypanosomiasis (HAT)] was performed and differences in the lipid, amino acid and metabolite profiles were identified. The most striking difference between the HAT patients and the control group was that six significantly altered amounts of LPC species were lower in patients than in controls (69). The interpretation of these data suggests that even though *T. brucei rhodesiense* is known to synthesize LPC *de novo*, the parasite probably scavenges several lipids from the host, as needed. Bearing in mind the results from our group on the key roles LPC play in *T. cruzi* infection, it is not surprising that LPC most likely engage crucial functions in other diseases caused by trypanosomatids.

### Schistosomiasis

The first connection between LPC and the biology of schistosomiasis was noticed by the modifications on lipid plasma composition in infected patients (70). Curiously, in the plasma of infected hamsters, the levels of LPC are similar in both control and infected patients but major differences were found in the composition of the fatty acid acyl chains (71). LPC fatty acid composition showed a decrease on 18:2 and 20:5, but less marked changes in 20:4 (71). Usually, human red blood cells (RBCs) are lysed by schistosomula of *Schistosoma mansoni*. LPC-derived from the parasite is used to lyse RBCs (72). The source of this LPC is probably the parasite itself once schistosomula synthesize LCP and release it in the culture medium as evaluated with experiments using [^3^H]-phospholipids (72). The dynamics of such process was further through the use of precursors to label phospholipid and neutral lipid with the use of acyl chains ([^3^H]palmitic acid and [^3^H]oleic acid) or phospholipid polar head groups ([^3^H]choline and [^14^C]ethanolamine) (73). Schistosomal-derived lipids and LPC were shown to stimulate macrophages *in vitro* and induced TLR2-dependent NF-κB activation and cytokine production (74). Administration of LPC induced eosinophil recruitment and cytokine production, in a mechanism largely dependent on TLR2. Thus, parasite-derived LPC mediated key events on the pathogenesis and lethality of schistosomiasis.

### Everywhere

The use of LPC on host × parasite interaction seems to be a ubiquous strategy to allow the handling of intracellular signaling pathways. Indeed, LPC may be a handful molecule also used by predators while hunting their daily meal. Snakes and bugs contain lysophospholipids in their saliva (75), *Belostoma anurum* saliva. This predator insect injects its saliva into the prey, which becomes immobilized (76). LPC isolated from saliva is neuroparalytic *in vitro* and *in vivo* (76), and it is highly likely that induce membrane depolarization and calcium entry into sensory nerves (76, 77). Indeed, LPC represents 25% of the total phospholipids present in the saliva of such insect. Since *Belostoma* employs extra-oral digestion LPC also helps in the digestion of preys larger than this insect such as amphibians and small fishes. Thus, it was shown that LPC have paralytic activity in zebrafish, which was the first evidence that lysophospholipids might play an important role in prey immobilization (76). Lysophospholipids and specially LPC paralyze the neuromuscular junction and are also present on snake venoms (78, 79). LPC can also modulate the activity of NK cells (79). Indeed, snake presynaptic phospholipase A2 neurotoxins are commonly present on snake venoms. Mass spectromic analysis of the lipids resulting from SPAN action demonstrated several forms of LPC including myristoyl-LPC and fatty acids (77). Altogether the above finding suggest that LPC use both as a immune and neuromodulator is an ancient biochemical and pharmacological use for such molecule, which probably arose from the eventual leak of modified phospholipids from membranes of interacting organisms whether in the mammalian × protozoan model or in the insect × protozoan model (80, 81).

## Conclusion

In the future, drugs that target LPC of the signaling systems conveying LPC-mediated signals may largely affect the transmission and pathogenesis of several of the above mentioned diseases. The development of such drugs has been hampered by the yet unknown true LPC receptor. A functional analysis of novel LPC receptor candidates and the test of hypothesis regarding the parasite receptor recently described will provide a wider view of the potential of such molecule to trigger investigations in this area. It is quite interesting that the potential possibility of LPC modulates the several steps of TLR-mediated signaling or the interaction of this cascade with downstream effectors. Due to its major role as a suppressor of NO production in the presence of LPS, it is likely that LPC mechanism of action provides a novel system to understand the functionality of still unknown transcriptional factors as well as the hierarchical mechanism of MAPK regulation. It is quite challenging to imagine that the previously known structural phospholipids are yet a potential source of yet not completed understood signaling modules. The seminal discover of lysophospholipid role in human atherosclerosis combined with its potential selection as an immunosupressor, pharmacological, and neurological modulator by bugs, parasites, worms, and snakes leads to an increase interest on how far the ubiquity of such molecules has been shaped during evolution. The detailed investigation of such models at the molecular level in the future will certainly provide in the near future several novel targets for blocking the transmission and pathogenesis of such diseases.

## Author Contributions

MS-N, AL, and GA discussed the data and wrote the paper. MS-N and GA are both the corresponding authors.

## Conflict of Interest Statement

The authors declare that the research was conducted in the absence of any commercial or financial relationships that could be construed as a potential conflict of interest.
